# Poly[[{μ_4_-2,2′-[butane-1,4-diylbis(sulfanedi­yl)]bis­(1,3,4-thia­diazole)}silver(I)] perchlorate sesquihydrate]

**DOI:** 10.1107/S1600536812022957

**Published:** 2012-05-31

**Authors:** Jia-Jia Li, Wei-Min Zhu

**Affiliations:** a College of Chemistry and Molecular Engineering, Zhengzhou University, Zhengzhou 450001, People’s Republic of China

## Abstract

In the polymeric title compound, {[Ag(C_8_H_10_N_4_S_4_)]ClO_4_·1.5H_2_O}_*n*_, the Ag^I^ atom has a slightly distorted trigonal-planar coordination geometry provided by three N-atom donors from the thia­diazole rings of three symmetry-related 2,2′-[butane-1,4-diylbis(sulfanedi­yl)]bis­(1,3,4-thia­diazole) ligands. Centrosymmetrically related Ag^I^ atoms are bridged by the N–N fragments of rings, forming six-membered dinuclear metallacycles, which are further linked by the alkyl spacers of the thia­diazole ligands into a layer network extending parallel to (0-21). The crystal structure is stabilized by inter­molecular O—H⋯O hydrogen bonds. The O atoms of the perchlorate anion and one water mol­ecule are disordered over two sets of sites with refined occupancy ratios of 0.640 (6):0.360 (6) and 0.663 (11):0.337 (11), respectively. The second water molecule shows half-occupancy.

## Related literature

For related polymeric Ag^I^ complexes, see: Yu *et al.* (2006 ▶); Wang & Ma (2007 ▶).
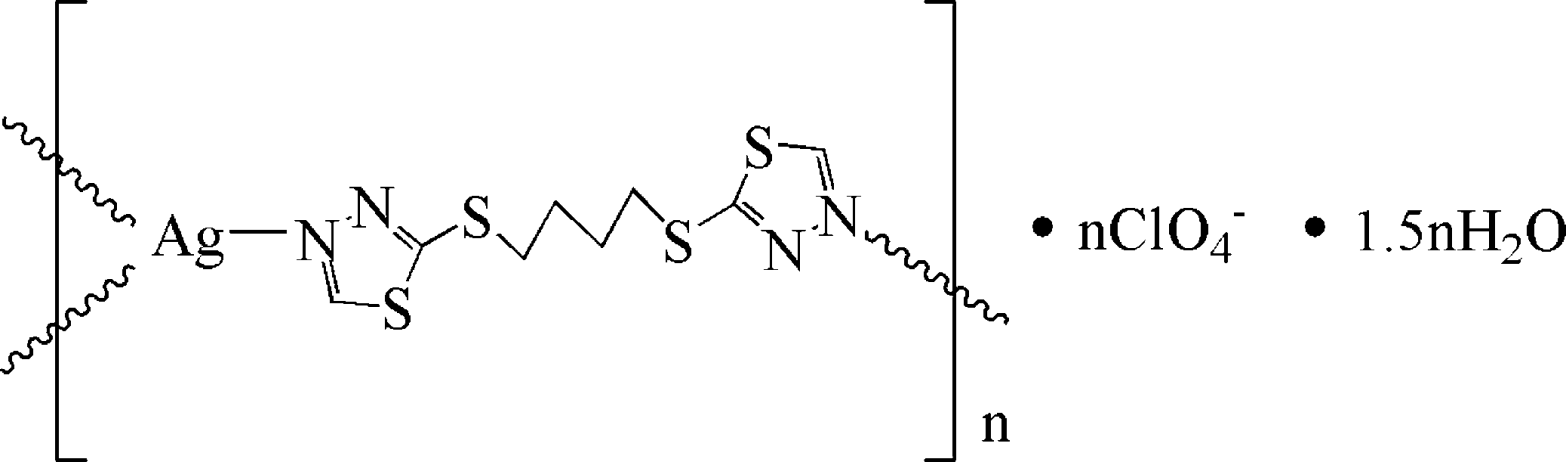


## Experimental

### 

#### Crystal data

[Ag(C_8_H_10_N_4_S_4_)]ClO_4_·1.5H_2_O
*M* *_r_* = 524.79
Triclinic, 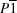

*a* = 10.076 (12) Å
*b* = 10.08 (2) Å
*c* = 10.137 (12) Å
α = 92.02 (2)°
β = 119.727 (14)°
γ = 94.20 (2)°
*V* = 889 (3) Å^3^
*Z* = 2
Mo *K*α radiation
μ = 1.78 mm^−1^
*T* = 291 K
0.29 × 0.04 × 0.04 mm

#### Data collection

Bruker SMART CCD area-detector diffractometer
Absorption correction: multi-scan (*SADABS*; Bruker, 1997 ▶) *T*_min_ = 0.624, *T*_max_ = 0.940
6708 measured reflections
3237 independent reflections
1834 reflections with *I* > 2σ(*I*)
*R*_int_ = 0.043

#### Refinement

*R*[*F* ^2^ > 2σ(*F* ^2^)] = 0.055
*wR*(*F* ^2^) = 0.143
*S* = 1.00
3237 reflections
214 parameters
236 restraints
H-atom parameters constrained
Δρ_max_ = 0.64 e Å^−3^
Δρ_min_ = −0.75 e Å^−3^

### 

Data collection: *SMART* (Bruker, 1997 ▶); cell refinement: *SAINT* (Bruker, 1997 ▶); data reduction: *SAINT*; program(s) used to solve structure: *SHELXS97* (Sheldrick, 2008 ▶); program(s) used to refine structure: *SHELXL97* (Sheldrick, 2008 ▶); molecular graphics: *SHELXTL* (Sheldrick, 2008 ▶); software used to prepare material for publication: *SHELXTL*.

## Supplementary Material

Crystal structure: contains datablock(s) I, global. DOI: 10.1107/S1600536812022957/rz2753sup1.cif

Structure factors: contains datablock(s) I. DOI: 10.1107/S1600536812022957/rz2753Isup2.hkl

Additional supplementary materials:  crystallographic information; 3D view; checkCIF report

## Figures and Tables

**Table 1:** Hydrogen-bond geometry (Å, °)

*D*—H⋯*A*	*D*—H	H⋯*A*	*D*⋯*A*	*D*—H⋯*A*
O6—H6*W*⋯O4′^i^	0.85	2.15	2.71 (3)	123
O6—H6*W*⋯O2^i^	0.85	1.61	2.46 (4)	178
O6—H5*W*⋯O1^ii^	0.85	2.47	3.02 (6)	123
O6—H5*W*⋯O6^iii^	0.85	2.36	3.10 (4)	146
O6—H5*W*⋯O4′^ii^	0.85	1.98	2.62 (6)	131
O5—H2*W*⋯O1^i^	0.85	2.42	3.27 (4)	179

Symmetry codes: (i) 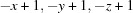; (ii) 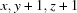; (iii) 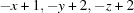.

**Fig. 1.**
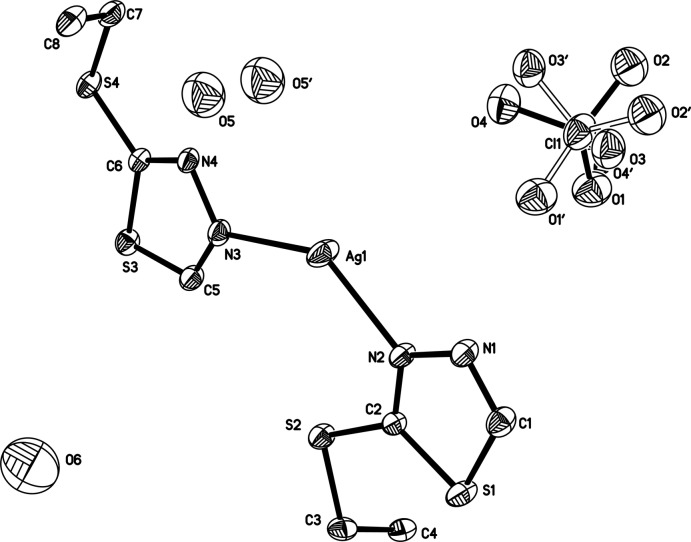
The asymmetric unit of the title compound, with displacement ellipsoids drawn at the 30% probability level. Hydrogen atoms are omitted.

**Fig. 2.**
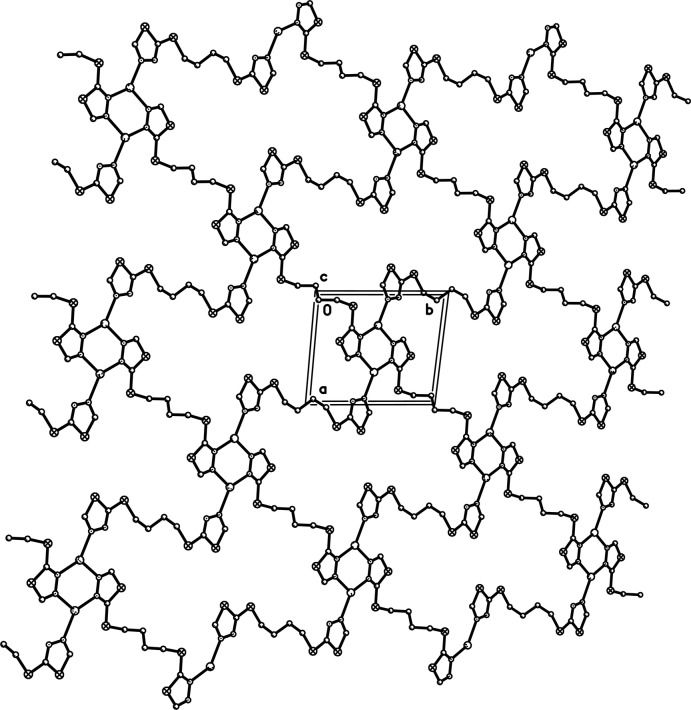
Partial crystal packing of the title compound showing the two-dimensional layer structure. Hydrogen atoms, water molecules and perchlorate anions are omitted.

## supplementary crystallographic information

### Comment

During the last decade, a great effort has been devoted to designing ligands capable of enforcing close metal contacts during the process of assembly or crystallization to form polynuclear complexes and coordination polymers, owing to these complexes are expected to produce specific structures, properties and reactivities, not found for mononuclear complexes. In particular, N,N'-linkage ligands such as 1,2-diazines, 1,2-diazoles, 1,2,4-triazoles and 1,3,4-thiadiazole are very versatile ligands that are able to bridge a wide range of intermetallic separations through two close adjacent N donors (Yu *et al.*, 2006; Wang & Ma, 2007).

In the title compound (Fig. 1), the Ag metal is coordinated by three N donors from thiadiazole rings of three distinct 2,2'-(butane-1,4-diyldithio)-bis(1,3,4-thiadiazole) ligands in a slightly distorted trigonal planar coordination geometry, with the metal protruding 0.0172 (15) Å from the N_3_ coordination plane. The Ag-N bond distances fall in the range 2.232 (8)-2.311 (9) Å. Centrosymmetrically related Ag metals are doubly bridged by the N-N fragments of thiadiazole rings of two distinct ligands to form six-membered dinuclear metallacycles. The Ag···Ag separation within the rings is 3.722 (4) Å, which is longer than the summed van der Waals radii of two free Ag ions (3.44 Å). The six-membered rings are linked by the alkyl spacers of the ligand into a two-dimensional layer network extending parallel to the (0 -2 1) plane (Fig. 2). Each Ag metal in the layer shows weak interactions with one S atom from an adjacent layer (Ag···S separation of 3.116 (5) Å), and with the oxygen atoms of the disordered ClO4^-^ anion, the shortest Ag···O separation being 2.816 (8) Å. The crystal structure is enforced by intermolecular O—H···O hydrogen bonds (Table 1).

### Experimental

The reaction of bis[2,2'-(butane-1,4-diyldithio)-bis(1,3,4-thiadiazole)] (0.1 mmol) with AgCl0_4_ (0.1 mmol) in MeOH (10 mL) for a few minutes afforded a light white solid, which was filtered, washed with acetone, and dried in air. Single crystals of the title compound suitable for X-ray analysis were obtained by slow diffusion of Et_2_O into an acetonitrile solution of the solid.

### Refinement

The oxygen atoms of the perchlorate anion and the water molecule including the O5 oxygen atoms are disordered over two sets of sites with refined site occupancy ratios of 0.640 (6):0.360 (6) and 0.663 (11):0.337 (11) respectively. The anisotropic displacement parameters for paired components of the disordered atoms were constrained to be equivalent and approximately isotropic by the EADP and ISOR commands in SHELXL-97 (Sheldrick, 2008). Water H atoms were located in a difference Fourier map and allowed to ride on the parent oxygen atoms, with O—H = 0.85 Å and with *U*_iso_(H) = 1.5 *U*_eq_(O). All other H atoms were positioned geometrically and refined as riding, with C—H = 0.93-0.97 Å and *U*_iso_(H) = 1.2*U*_eq_(C).

### Crystal data

**Table:**

[Ag(C_8_H_10_N_4_S_4_)]ClO_4_·1.5H_2_O	*Z* = 2
*M**_r_* = 524.79	*F*(000) = 522
Triclinic, *P*1	*D*_x_ = 1.961 Mg m^−^^3^
Hall symbol: -P 1	Mo *K*α radiation, λ = 0.71073 Å
*a* = 10.076 (12) Å	Cell parameters from 954 reflections
*b* = 10.08 (2) Å	θ = 2.3–19.5°
*c* = 10.137 (12) Å	µ = 1.78 mm^−^^1^
α = 92.02 (2)°	*T* = 291 K
β = 119.727 (14)°	Block, colourless
γ = 94.20 (2)°	0.29 × 0.04 × 0.04 mm
*V* = 889 (3) Å^3^	

### Data collection

**Table:**

Bruker SMART CCD area-detector diffractometer	3237 independent reflections
Radiation source: fine-focus sealed tube	1834 reflections with *I* > 2σ(*I*)
Graphite monochromator	*R*_int_ = 0.043
phi and ω scans	θ_max_ = 25.5°, θ_min_ = 2.9°
Absorption correction: multi-scan (*SADABS*; Bruker, 1997)	*h* = −12→12
*T*_min_ = 0.624, *T*_max_ = 0.940	*k* = −12→11
6708 measured reflections	*l* = −12→12

### Refinement

**Table:**

Refinement on *F*^2^	Primary atom site location: structure-invariant direct methods
Least-squares matrix: full	Secondary atom site location: difference Fourier map
*R*[*F*^2^ > 2σ(*F*^2^)] = 0.055	Hydrogen site location: inferred from neighbouring sites
*wR*(*F*^2^) = 0.143	H-atom parameters constrained
*S* = 1.00	*w* = 1/[σ^2^(*F*_o_^2^) + (0.0596*P*)^2^ + 0.9001*P*] where *P* = (*F*_o_^2^ + 2*F*_c_^2^)/3
3237 reflections	(Δ/σ)_max_ < 0.001
214 parameters	Δρ_max_ = 0.64 e Å^−^^3^
236 restraints	Δρ_min_ = −0.75 e Å^−^^3^

### Special details

**Table:**

Geometry. All esds (except the esd in the dihedral angle between two l.s. planes) are estimated using the full covariance matrix. The cell esds are taken into account individually in the estimation of esds in distances, angles and torsion angles; correlations between esds in cell parameters are only used when they are defined by crystal symmetry. An approximate (isotropic) treatment of cell esds is used for estimating esds involving l.s. planes.
Refinement. Refinement of F^2^ against ALL reflections. The weighted R-factor wR and goodness of fit S are based on F^2^, conventional R-factors R are based on F, with F set to zero for negative F^2^. The threshold expression of F^2^ > 2sigma(F^2^) is used only for calculating R-factors(gt) etc. and is not relevant to the choice of reflections for refinement. R-factors based on F^2^ are statistically about twice as large as those based on F, and R- factors based on ALL data will be even larger.

### Fractional atomic coordinates and isotropic or equivalent isotropic displacement parameters (Å^2^)

**Table:**

	*x*	*y*	*z*	*U*_iso_*/*U*_eq_	Occ. (<1)
O5	0.5870 (14)	0.9980 (12)	0.5479 (13)	0.111 (3)	0.663 (11)
H1W	0.5826	0.9510	0.4743	0.166*	0.663 (11)
H2W	0.5493	0.9603	0.5974	0.166*	0.663 (11)
O5'	0.709 (3)	0.984 (2)	0.599 (3)	0.111 (3)	0.337 (11)
H3W	0.6772	0.9410	0.5137	0.166*	0.337 (11)
H4W	0.6737	0.9609	0.6564	0.166*	0.337 (11)
Cl1	0.6364 (2)	0.2700 (2)	0.2629 (3)	0.0868 (8)	0.640 (6)
O1	0.5621 (6)	0.1488 (5)	0.2654 (8)	0.105 (2)	0.640 (6)
O2	0.7031 (8)	0.2400 (7)	0.1638 (8)	0.107 (2)	0.640 (6)
O3	0.7646 (7)	0.3177 (6)	0.4046 (6)	0.093 (2)	0.640 (6)
O4	0.5392 (6)	0.3695 (5)	0.1966 (8)	0.098 (2)	0.640 (6)
Cl1'	0.6364 (2)	0.2700 (2)	0.2629 (3)	0.0868 (8)	0.360 (6)
O1'	0.5900 (8)	0.3025 (7)	0.3739 (8)	0.105 (2)	0.360 (6)
O2'	0.7956 (5)	0.2706 (6)	0.3363 (8)	0.107 (2)	0.360 (6)
O3'	0.5824 (7)	0.3645 (6)	0.1525 (7)	0.093 (2)	0.360 (6)
O4'	0.5605 (7)	0.1401 (5)	0.1931 (7)	0.098 (2)	0.360 (6)
Ag1	0.28732 (7)	0.49669 (7)	0.38379 (8)	0.0710 (3)	
S1	0.4338 (2)	0.2152 (2)	0.7857 (2)	0.0592 (6)	
S2	0.1184 (2)	0.28824 (19)	0.5359 (2)	0.0517 (5)	
S3	−0.2397 (2)	0.5507 (2)	0.0592 (2)	0.0525 (5)	
S4	−0.1933 (2)	0.74107 (19)	−0.1386 (2)	0.0539 (5)	
N1	0.5493 (7)	0.3652 (6)	0.6644 (7)	0.0519 (16)	
N2	0.3926 (6)	0.3663 (6)	0.5770 (6)	0.0464 (15)	
N3	0.0497 (7)	0.5526 (6)	0.2226 (6)	0.0488 (15)	
N4	0.0286 (6)	0.6374 (5)	0.1089 (6)	0.0433 (14)	
C1	0.5838 (9)	0.2893 (8)	0.7730 (9)	0.057 (2)	
H1	0.6850	0.2755	0.8411	0.069*	
C2	0.3171 (8)	0.2935 (7)	0.6263 (8)	0.0424 (17)	
C3	0.0689 (9)	0.1618 (8)	0.6326 (9)	0.054 (2)	
H3A	0.1430	0.1754	0.7408	0.065*	
H3B	−0.0309	0.1755	0.6198	0.065*	
C4	0.0641 (9)	0.0183 (8)	0.5792 (8)	0.055 (2)	
H4A	0.1607	0.0053	0.5839	0.065*	
H4B	0.0524	−0.0404	0.6473	0.065*	
C5	−0.0794 (9)	0.5012 (8)	0.2069 (9)	0.0518 (19)	
H5	−0.0839	0.4410	0.2724	0.062*	
C6	−0.1156 (8)	0.6454 (6)	0.0185 (7)	0.0401 (16)	
C7	−0.0201 (9)	0.8336 (8)	−0.1127 (9)	0.060 (2)	
H7A	0.0559	0.7718	−0.0924	0.072*	
H7B	−0.0431	0.8718	−0.2073	0.072*	
C8	0.0493 (10)	0.9440 (8)	0.0136 (10)	0.068 (2)	
H8A	0.0685	0.9068	0.1077	0.082*	
H8B	0.1475	0.9798	0.0268	0.082*	
O6	0.337 (2)	0.974 (2)	0.981 (2)	0.169 (8)	0.50
H5W	0.4339	0.9960	1.0303	0.254*	0.50
H6W	0.3215	0.9007	0.9296	0.254*	0.50

### Atomic displacement parameters (Å^2^)

**Table:**

	*U*^11^	*U*^22^	*U*^33^	*U*^12^	*U*^13^	*U*^23^
O5	0.104 (6)	0.128 (6)	0.102 (6)	0.027 (5)	0.051 (5)	0.015 (5)
O5'	0.104 (6)	0.128 (6)	0.102 (6)	0.027 (5)	0.051 (5)	0.015 (5)
Cl1	0.0565 (13)	0.0827 (16)	0.0970 (17)	0.0042 (11)	0.0195 (12)	0.0245 (14)
O1	0.099 (4)	0.099 (4)	0.109 (4)	−0.007 (4)	0.048 (3)	0.013 (4)
O2	0.084 (4)	0.116 (5)	0.110 (4)	0.006 (3)	0.041 (3)	0.010 (4)
O3	0.079 (4)	0.104 (4)	0.087 (4)	0.023 (3)	0.033 (3)	0.010 (3)
O4	0.084 (4)	0.092 (4)	0.105 (5)	0.018 (3)	0.035 (3)	0.011 (3)
Cl1'	0.0565 (13)	0.0827 (16)	0.0970 (17)	0.0042 (11)	0.0195 (12)	0.0245 (14)
O1'	0.099 (4)	0.099 (4)	0.109 (4)	−0.007 (4)	0.048 (3)	0.013 (4)
O2'	0.084 (4)	0.116 (5)	0.110 (4)	0.006 (3)	0.041 (3)	0.010 (4)
O3'	0.079 (4)	0.104 (4)	0.087 (4)	0.023 (3)	0.033 (3)	0.010 (3)
O4'	0.084 (4)	0.092 (4)	0.105 (5)	0.018 (3)	0.035 (3)	0.011 (3)
Ag1	0.0421 (4)	0.0867 (5)	0.0724 (5)	0.0093 (3)	0.0171 (3)	0.0441 (4)
S1	0.0534 (12)	0.0638 (14)	0.0507 (12)	0.0034 (10)	0.0179 (10)	0.0237 (10)
S2	0.0405 (10)	0.0521 (12)	0.0588 (12)	0.0008 (9)	0.0218 (9)	0.0155 (10)
S3	0.0385 (10)	0.0540 (12)	0.0567 (12)	0.0057 (9)	0.0173 (9)	0.0089 (10)
S4	0.0499 (11)	0.0509 (12)	0.0459 (11)	0.0124 (9)	0.0111 (9)	0.0139 (10)
N1	0.043 (4)	0.051 (4)	0.055 (4)	0.004 (3)	0.019 (3)	0.016 (3)
N2	0.037 (3)	0.047 (4)	0.048 (4)	0.003 (3)	0.015 (3)	0.017 (3)
N3	0.045 (4)	0.051 (4)	0.045 (4)	0.016 (3)	0.017 (3)	0.011 (3)
N4	0.037 (3)	0.039 (3)	0.050 (4)	0.009 (3)	0.018 (3)	0.011 (3)
C1	0.045 (4)	0.058 (5)	0.057 (5)	0.002 (4)	0.016 (4)	0.013 (4)
C2	0.043 (4)	0.043 (4)	0.040 (4)	0.005 (3)	0.020 (3)	0.007 (3)
C3	0.050 (5)	0.060 (5)	0.055 (5)	−0.007 (4)	0.029 (4)	0.009 (4)
C4	0.058 (5)	0.054 (5)	0.051 (4)	−0.001 (4)	0.027 (4)	0.012 (4)
C5	0.052 (5)	0.048 (5)	0.053 (5)	0.011 (4)	0.024 (4)	0.014 (4)
C6	0.039 (4)	0.034 (4)	0.038 (4)	0.004 (3)	0.013 (3)	0.005 (3)
C7	0.073 (6)	0.054 (5)	0.053 (5)	0.019 (4)	0.029 (4)	0.020 (4)
C8	0.067 (6)	0.051 (5)	0.076 (6)	0.008 (4)	0.028 (5)	0.021 (5)
O6	0.162 (11)	0.181 (11)	0.168 (11)	0.007 (8)	0.087 (8)	0.007 (8)

### Geometric parameters (Å, º)

**Table:**

O5—O5^i^	1.53 (2)	S4—C6	1.750 (7)
O5—H1W	0.8502	S4—C7	1.810 (8)
O5—H2W	0.8498	N1—C1	1.280 (9)
O5—H3W	1.2854	N1—N2	1.376 (8)
O5—H4W	1.1129	N1—Ag1^ii^	2.323 (6)
O5'—H1W	1.2852	N2—C2	1.300 (8)
O5'—H3W	0.8500	N3—C5	1.296 (9)
O5'—H4W	0.8500	N3—N4	1.398 (8)
Cl1—O1	1.393 (5)	N4—C6	1.288 (8)
Cl1—O4	1.398 (5)	C1—H1	0.9300
Cl1—O3	1.408 (4)	C3—C4	1.516 (11)
Cl1—O2	1.494 (5)	C3—H3A	0.9700
Cl1'—O2'	1.394 (4)	C3—H3B	0.9700
Cl1'—O3'	1.415 (5)	C4—C4^iii^	1.487 (14)
Cl1'—O4'	1.433 (5)	C4—H4A	0.9700
Cl1'—O1'	1.452 (4)	C4—H4B	0.9700
Ag1—N2	2.239 (6)	C5—H5	0.9300
Ag1—N3	2.259 (6)	C7—C8	1.505 (11)
Ag1—N1^ii^	2.323 (6)	C7—H7A	0.9700
S1—C1	1.701 (8)	C7—H7B	0.9700
S1—C2	1.720 (7)	C8—C8^iv^	1.501 (15)
S2—C2	1.736 (7)	C8—H8A	0.9700
S2—C3	1.821 (7)	C8—H8B	0.9700
S3—C5	1.695 (8)	O6—H5W	0.8500
S3—C6	1.735 (7)	O6—H6W	0.8498
			
O5^i^—O5—H1W	88.4	C6—N4—N3	110.5 (5)
O5^i^—O5—H2W	74.1	N1—C1—S1	116.1 (6)
H1W—O5—H2W	116.5	N1—C1—H1	122.0
O5^i^—O5—H3W	129.1	S1—C1—H1	122.0
H2W—O5—H3W	121.9	N2—C2—S1	113.4 (5)
O5^i^—O5—H4W	139.9	N2—C2—S2	119.8 (5)
H1W—O5—H4W	108.3	S1—C2—S2	126.8 (4)
H2W—O5—H4W	65.8	C4—C3—S2	115.7 (6)
H3W—O5—H4W	75.3	C4—C3—H3A	108.4
H1W—O5'—H4W	94.7	S2—C3—H3A	108.4
H3W—O5'—H4W	120.0	C4—C3—H3B	108.4
O1—Cl1—O4	114.6	S2—C3—H3B	108.4
O1—Cl1—O3	114.0	H3A—C3—H3B	107.4
O4—Cl1—O3	111.4	C4^iii^—C4—C3	112.3 (8)
O1—Cl1—O2	105.0	C4^iii^—C4—H4A	109.1
O4—Cl1—O2	106.5	C3—C4—H4A	109.1
O3—Cl1—O2	104.3	C4^iii^—C4—H4B	109.1
O2'—Cl1'—O3'	112.2	C3—C4—H4B	109.1
O2'—Cl1'—O4'	111.4	H4A—C4—H4B	107.9
O3'—Cl1'—O4'	109.7	N3—C5—S3	115.6 (6)
O2'—Cl1'—O1'	109.6	N3—C5—H5	122.2
O3'—Cl1'—O1'	107.8	S3—C5—H5	122.2
O4'—Cl1'—O1'	105.9	N4—C6—S3	115.6 (5)
N2—Ag1—N3	136.8 (2)	N4—C6—S4	125.7 (5)
N2—Ag1—N1^ii^	118.1 (2)	S3—C6—S4	118.7 (4)
N3—Ag1—N1^ii^	104.8 (2)	C8—C7—S4	115.0 (6)
C1—S1—C2	86.5 (4)	C8—C7—H7A	108.5
C2—S2—C3	102.5 (3)	S4—C7—H7A	108.5
C5—S3—C6	86.1 (4)	C8—C7—H7B	108.5
C6—S4—C7	100.3 (3)	S4—C7—H7B	108.5
C1—N1—N2	111.0 (6)	H7A—C7—H7B	107.5
C1—N1—Ag1^ii^	128.6 (5)	C8^iv^—C8—C7	114.0 (9)
N2—N1—Ag1^ii^	120.1 (4)	C8^iv^—C8—H8A	108.8
C2—N2—N1	113.0 (6)	C7—C8—H8A	108.8
C2—N2—Ag1	125.1 (5)	C8^iv^—C8—H8B	108.8
N1—N2—Ag1	121.7 (4)	C7—C8—H8B	108.8
C5—N3—N4	112.3 (6)	H8A—C8—H8B	107.6
C5—N3—Ag1	127.3 (5)	H5W—O6—H6W	107.3
N4—N3—Ag1	119.7 (4)		
			
C1—N1—N2—C2	1.4 (9)	N1—N2—C2—S2	177.2 (5)
Ag1^ii^—N1—N2—C2	−173.9 (5)	Ag1—N2—C2—S2	2.4 (9)
C1—N1—N2—Ag1	176.4 (5)	C1—S1—C2—N2	−0.4 (6)
Ag1^ii^—N1—N2—Ag1	1.1 (7)	C1—S1—C2—S2	−177.8 (5)
N3—Ag1—N2—C2	1.4 (8)	C3—S2—C2—N2	174.1 (6)
N1^ii^—Ag1—N2—C2	173.3 (6)	C3—S2—C2—S1	−8.6 (6)
N3—Ag1—N2—N1	−173.0 (4)	C2—S2—C3—C4	−80.2 (6)
N1^ii^—Ag1—N2—N1	−1.0 (7)	S2—C3—C4—C4^iii^	−67.4 (10)
N2—Ag1—N3—C5	−8.1 (8)	N4—N3—C5—S3	−1.7 (8)
N1^ii^—Ag1—N3—C5	179.3 (6)	Ag1—N3—C5—S3	−172.0 (3)
N2—Ag1—N3—N4	−177.8 (4)	C6—S3—C5—N3	1.0 (6)
N1^ii^—Ag1—N3—N4	9.6 (5)	N3—N4—C6—S3	−0.8 (8)
C5—N3—N4—C6	1.6 (8)	N3—N4—C6—S4	179.6 (5)
Ag1—N3—N4—C6	172.7 (5)	C5—S3—C6—N4	0.0 (6)
N2—N1—C1—S1	−1.8 (9)	C5—S3—C6—S4	179.6 (5)
Ag1^ii^—N1—C1—S1	173.0 (4)	C7—S4—C6—N4	−7.9 (7)
C2—S1—C1—N1	1.3 (7)	C7—S4—C6—S3	172.5 (4)
N1—N2—C2—S1	−0.5 (8)	C6—S4—C7—C8	−74.4 (6)
Ag1—N2—C2—S1	−175.2 (3)	S4—C7—C8—C8^iv^	−65.6 (11)

Symmetry codes: (i) −*x*+1, −*y*+2, −*z*+1; (ii) −*x*+1, −*y*+1, −*z*+1; (iii) −*x*, −*y*, −*z*+1; (iv) −*x*, −*y*+2, −*z*.

### Hydrogen-bond geometry (Å, º)

**Table:**

*D*—H···*A*	*D*—H	H···*A*	*D*···*A*	*D*—H···*A*
O6—H6*W*···O4′^ii^	0.85	2.15	2.71 (3)	123
O6—H6*W*···O2^ii^	0.85	1.61	2.46 (4)	178
O6—H5*W*···O1^v^	0.85	2.47	3.02 (6)	123
O6—H5*W*···O6^vi^	0.85	2.36	3.10 (4)	146
O6—H5*W*···O4′^v^	0.85	1.98	2.62 (6)	131
O5—H2*W*···O1^ii^	0.85	2.42	3.27 (4)	179

Symmetry codes: (ii) −*x*+1, −*y*+1, −*z*+1; (v) *x*, *y*+1, *z*+1; (vi) −*x*+1, −*y*+2, −*z*+2.
